# Prophylaxis of heterotopic ossification – an updated review

**DOI:** 10.1186/1749-799X-4-12

**Published:** 2009-04-20

**Authors:** Evan O Baird, Qian K Kang

**Affiliations:** 1 Clemson University, Department of Bioengineering, Clemson, SC, USA; 2 Medical University of South Carolina, Department of Orthopaedic Surgery, Charleston, SC, USA

## Abstract

Heterotopic ossification (HO) is defined as the process by which trabecular bone forms outside of the skeletal structure, occupying space in soft tissue where it does not normally exist. The current popular prophylactic treatment modalities include non-steroidal anti-inflammatory drugs (NSAIDs) and radiation therapy, although the literature remains inconclusive as to which is superior. Additionally, both treatments can lead to adverse effects to the patient. Recently there have been several studies attempting to identify new aspects of the etiology of heterotopic bone formation and introduce new prophylactic modalities with increased efficacy and fewer side effects. For this review, we selectively retrieved articles from Medline published from 1958–2008 on the prophylaxis of HO with the aim of assisting readers in quickly grasping the current status of research and clinical aspects of HO prophylaxis.

## Background

Heterotopic ossification (HO) is defined as the process by which trabecular bone forms outside of the skeletal structure, occupying space in soft tissue where it does not normally exist. This misplaced growth occurs between muscle planes and not within the muscle fibers themselves. Furthermore, though the new bone often abuts existing skeletal structure, it does not interfere with the configuration of the periosteum[1].

The heterotopic ossification of muscles, ligaments and tendons is a potential complication following trauma, elective surgery, neurological injury and severe burns[2]. The most common site for the formation of HO is following open-reduction internal-fixation (ORIF) for acetabular fracture, followed by the hip after total hip arthroplasty (THA)[3,4]. Following THA, the incidence of HO has been reported as being between 5 and 90%, though only 3 to 7% of patients experience clinically significant HO; that is, to an extent that the outcome of the surgery is affected and as designated a grade of III or IV as originally described by Brooker in reference to the hip joint (Table 1)[5,6]. Reported incidence is lower following primary total knee arthroplasty (TKA), and has been reported as between 3.8 and 39% for all Brooker classification grades, but as one study reported only 1% of patients were symptomatic[5].

**Table 1:** Brooker classification of heterotopic ossification [6]

Grade 1	Islands of bone within the soft tissues about the hip
Grade 2	Bone spurs from the pelvis or proximal end of the femur, leaving at least one centimeter between opposing bone surfaces
Grade 3	Bone spurs from the pelvis or proximal end of the femur, reducing the space between opposing bone surfaces to less than one centimeter
Grade 4	Apparent bone ankylosis of the hip

This article is meant to serve as a review and an update on the literature regarding current prophylaxis modalities as well as treatments under investigation for prevention of heterotopic ossification. It is our hope that these new methods will be more effective and with fewer side effects than their predecessors, allowing far fewer patients to suffer with the debilitating effects of this disease.

## Mechanisms of heterotopic ossification

The etiology of HO can be divided into the three headings of neurological, genetic and traumatic, with orthopaedic procedures included in this last group[5]. Though the etiology has been classified, the exact pathophysiology of HO remains unknown. Several contributory factors have been suggested. Prostaglandin activity, specifically PGE-2, as well as hypercalcemia, tissue hypoxia, alterations in sympathetic nerve activity, prolonged immobilization and imbalances between parathyroid hormone activity and calcitonin have all been shown to contribute to HO formation[7]. These factors help to enable the improper differentiation of pluripotent mesenchymal stem cells into osteoblastic precursors[5]. Furthermore, numerous risk factors have been identified in several studies. Those studied in association with hip arthroplasty include a history of HO in the ipsilateral or contralateral hip, posttraumatic arthritis, hypertrophic osteoarthritis, rheumatoid arthritis, ankylosing spondylitis, diffuse idiopathic skeletal hyperostosis, osteonecrosis, Paget disease and male sex[8,9].

It is known that heterotopic ossification is determined by both systemic factors and by local tissue changes, primarily characterized by inflammation[10], cell death[11], and upregulation of mineralization growth factors [12-14]. Both inflammation and cell death can result in local alterations in pH and matrix that promote the deposition of calcium in the form of hydroxyapatite crystals. Growth factors such as BMPs stimulate mineralization and bone formation via their effects on osteoblast progenitor cells.

Several studies have utilized animal models as a means of studying the process and prevention of HO. These have included the use of rabbits, mice, rats, canines, goats and sheep and have had a significant impact on the study of HO[2,12-17]. The three most commonly used models are the rabbit quadriceps model, the rabbit hip model and the rodent soft tissue model with BMP injection. The rabbit quadriceps exercise model was developed by Michelsson et al[14]. One of the animal's legs is immobilized with the knee in extension using a plastic splint for up to 5 weeks. Six days a week, a 5 minute session of forceful mobilization through the full range of motion is performed. While Michelsson et al. previously[18] showed that immobilization in extension led to degenerative joint changes and stiffness of the joint that gradually resolved at the end of the immobilization period, forceful manipulation was required to result in permanent stiffness of the knee and development of heterotopic bone in the quadriceps muscles. This model reflects the clinical experience of patients with early immobilization followed by aggressive physical therapies[14]. A newer rabbit model involves the reaming of the femoral medullary canal through a greater trochanter approach, similar to that used in total hip arthroplasty [16]. Ischemic injury to the abductor musculature is induced by occlusion of vessels for 3 minutes during the procedure. Ischemia, as will be noted later in this manuscript, has been shown to create free-radicals in skeletal muscle tissue, causing injury to the tissue and as suggested by Vanden Bossche et al, may lead to the formation of HO[1]. This model creates HO in the abductor muscles which mimics the scenario of HO after total hip arthroplasty and acetabular fractures[16]. A third model uses bone morphogenetic proteins (BMPs) placed in skeletal muscle to induce HO[2]. These proteins have been demonstrated to induce HO in several previous studies[2,19-22]. Bone morphogenetic proteins have been shown to be present during fracture repair; furthermore, inhibition of chondrogenesis by transforming growth factor-beta has been shown to be overcome by addition of BMPs in murine models[23]. Moreover, cell culture models have demonstrated that BMPs initiate and maintain chondrocytes, and even appear to induce differentiation of osteoblast precursor cells into more mature osteoblast-like cells[23]. A collagen BMP-4-expressing muscle-derived stem cell impregnated sponge is inserted into the hamstring muscles of the rodent to induce HO[2].

The understanding of the process of induction of HO, the mediators involved (including the origin of involved osteoprogenitor cells[16]) and the reasoning behind prophylactic treatments in humans owe their origins to the observation of the phenomenon in animals. It is the opinion of the authors that the future direction of study will see animal models continuing to play a dominant role in advancing the understanding, treatment and prevention of HO.

## Commonly used prophylactic modalities

### NSAIDs

The prophylactic effectiveness of indomethacin following elective lower limb surgery is well-accepted, and has been shown to help prevent HO, lessen the extent of the development of HO and assist in preventing inflammation associated with acute HO; it is currently part of a widely-employed prophylaxis protocol[1,5,24-27]. The drug is a simple and low cost option for prophylaxis. It is important to note that several trials have shown that aspirin does not appear to have the same beneficial effects as indomethacin[27,28]. The drug works by inhibiting prostaglandin-mediated (specifically PGE-2) bone remodeling and also by directly inhibiting the differentiation of osteoprogenitor cells[1,24,25]. A Cochrane Review was conducted in 2004, comprising 16 randomized controlled trials analyzing the effectiveness of prevention of HO with the use of NSAIDs[28]. The use of NSAIDs peri-operatively was shown to reduce the risk of developing heterotopic bone formation by 59% over placebo. Another systematic review by Neal, et al. demonstrated a 57% reduction in the risk of HO when NSAIDs were used as prophylaxis[27]. The statistical data from this study leads the authors to predict that for every 100,000 THAs performed in the US each year, perioperative NSAID use has the potential to prevent anywhere from 10 to 20,000 cases of heterotopic bone formation. Further, the review showed no significant difference in the incidence of gastro-intestinal complications between those treated with NSAIDs and the control. Though both review studies demonstrated a significant reduction in incidence of HO, neither addressed clinical outcomes of pain and physical function among treatments.

However not all trials have seen such a benign side-effect profile with the use of NSAIDs. Karunakar et al[26]. conducted a randomized, prospective double-blind placebo-controlled clinical trial on the effect of indomethacin after the operative treatment of fractures of the acetabulum. Though as the authors point out the study is lacking in power, it is significant in that it was determined that there was no significant difference in the incidence of HO between the indomethacin and placebo treatment groups. However, it was noted that one of the indomethacin patients developed gastrointestinal hemorrhage and one had a perforated ulcer, while 13 total patients withdrew from the study due to side effects of the medication, compared with only 1 withdrawing in the placebo treatment. Others such as Banovac[24] have used misoprostol to aid in the prevention of gastrointestinal complications. Another problem with non-selective (ie those actively inhibiting cyclo-oxygenase 1 and 2, or COX-1 and COX-2) NSAIDs such as indomethacin is increased perioperative bleeding secondary to inhibition of COX-1, leading to reduced production of thromboxane A2, which is essential to platelet aggregation[29]. Fransen et al. followed up her 2004 Cochrane review with a randomized controlled trial comparing postoperative pain and physical function in patients taking either ibuprofen or placebo following total hip arthroplasty and revision total hip arthroplasty surgery[30]. Though the overall risk of HO was reduced by 31% (as assessed radiographically) with use of ibuprofen, the study demonstrated no clinically significant difference in either pain or physical function 6 to 12 months postoperatively. Further, though the risk reduction is impressive, the raw data (ie number of patients affected) does not suggest as large of an impact on the actual incidence of Brooker Grade 3 and 4 HO. Among those patients in the ibuprofen treatment set, 2.5% developed Brooker Grade 3 or 4 HO, whereas the placebo treatment demonstrated an incidence of 6%[30]. Further, there was a significant increase in major bleeding complications in the ibuprofen treatment group, again serving to emphasize untoward side effects of this treatment modality[30].

One method of circumventing the pitfalls of NSAIDs use is by employing COX-2 selective NSAIDs, such as meloxicam. Weber et al. have shown that use of this selective NSAID following total hip arthroplasty was associated with a 17% reduction in intraoperative and postoperative (first 24 hours following surgery) blood loss[29]. Furthermore, fewer gastrointestinal side-effects have been noted as a result of preferential use of COX-2 selective NSAIDs [31-35]. As noted in additional File 1, more recent studies have highlighted the both these advantages as well as efficacy comparable to that of traditional, non-selective NSAIDs[32,34-37]. However, it is notable that the practice of prescribing COX-2 specific NSAIDs is not without consequences, as several trials have shown an increased risk of cardiovascular events associated with their use [38-40]. Despite this, a few trials have shown either no increased risk for cardiovascular events when compared to non-slective NSAID use[41,42] (albeit with lower rates of GI side effects) or a decreased risk (in the case of celecoxib[43]) as shown in one study. Due to the current lack of evidence for safety of routine use of COX-2 selective NSAIDs as prophylaxis for postoperative HO, indomethacin remains the gold standard of treatment when employing NSAIDs therapy[44].

A serious problem with the use of high-dose indomethacin and other NSAIDS for HO prophylaxis is that while new heterotopic bone may be prevented from forming, the formation of bone for healing the fracture site may also be impaired. Thus, the large number of reports of nonunion or malunion as well as poor ligament healing[5,25,45]. One such study by Burd et al. noted 29% incidence of nonunion of long bone fractures following indomethacin prophylaxis, whereas in the radiation arm the incidence was just 7%[25]. Of note, there were no instances of acetabular nonunion. In a study by Persson et al., 142 patients were followed for formation of HO following THA. Of the 11 that underwent a revision procedure secondary to aseptic loosening, 10 belonged to the indomethacin group[46].

### Radiation Therapy

Studies by Cooley and Goss[47] in 1958 and later those by Craven and Urist[48] in 1971 demonstrated the effects of irradiation therapy on bone growth and repair. After demonstrating the inhibiting influence of the radiation on bone repair of rat bone and seeing that the effects were more pronounced when the treatment was initiated closer to the time of the fracture, the authors hypothesized that the early osteoprogenitor cells involved in bone repair were more radiosensitive than the more mature cells seen later[4]. In 1981, Coventry et al. established the utility of radiotherapy (RT) as prophylactic treatment for HO by irradiating the hips of 42 patients who had undergone hip surgery[49] 48 hips, each designated as high risk for the formation of HO, were treated with a 20 Gy dose of radiation. Of those treated, 19% developed ectopic bone, noting that patients treated earlier enjoyed lower rates of HO, though data specifics were not included. Today, RT is used prophylactically (albeit in much smaller doses) both pre- and postoperatively for the prevention of HO following bone fracture or manipulation secondary to trauma or operative treatment[3,4,50]. Recently, Childs et al. noted that based on a retrospective cohort study of 263 patients having experienced traumatic acetabular fracture, HO was discovered in 5.3% of patients receiving RT, while 60% of patients who did not receive treatment developed some degree of ectopic bone[51]. Further, a study by Chao et al. has demonstrated RT to be capable of preventing HO in high-risk patients, specifically those in whom a history of HO exists[52].

Several studies comparing preoperative to postoperative radiation therapy (RT) were reviewed by Balboni et al. in a critical review of RT for HO prophylaxis[4]. Studies cited by the authors suggest that there is not a statistically significant difference between employing preoperative (<4 hours preoperatively) or postoperative (<72 hours postoperatively) RT. In particular the authors cite Gregoritch et al[50] and Seegenschmiedt et al[53] In Gregoritch's study 122 patients undergoing THA and deemed at a high risk of HO were treated with either preoperative or postoperative RT. The authors reported no significant difference among the treatment arms, noting a 28% incidence in the postoperative treatment and 26% incidence in the preoperative treatment (*p *= *1.0*). They calculated a 5% incidence of clinically significant (Brooker Grade 3 or 4) HO in the postoperative group and a 2% incidence in the preoperative group (*p *= *0.58*). In Seegenschmiedt's study, 161 patients were randomized to receive either preoperative or postoperative RT. Of those treated, 4 (5%) failures were noted in the postoperative group, compared with 11 (19%) in the preoperative group (*p *<*0.05*). However, the authors noted that the majority of failures in the preoperative group occurred in patients with preexisting Brooker Grade 3 or 4 HO which had not been removed prior to treatment. Thus, the authors conclude that there both pre- and postoperative treatment are equally effective.

Childs et al. had similar results regarding timing of treatment in a study evaluating postoperative RT for HO prophylaxis following traumatic acetabular fractures[51]. Of 152 patients studied, 58 received radiation within 24 hours of surgery, 41 within 48 hours, 53 within 72 hours, 13 within 4 days and 4 were delayed longer than 4 days. The authors noted no increase in HO when prophylactic RT was initiated anywhere from one to four days postoperatively.

There are several potential side effects of RT, the most concerning of which is the theoretical effect of carcinogenesis; however there has yet to be a documented case of a radiation-induced tumor after RT for HO prophylaxis[4]. This positive outcome is thought to be the effect of both low doses of radiation as well as an older patient population – as the latency period for induction of malignancy following RT is from 15 to 24 years, it is a possibility that there are too few patients that survive long enough after treatment for the carcinogenic effects to be realized[4,50]. Therefore, as this treatment option is employed for younger patients, this concern is worth considering.

Another possible complication of RT (and one that also plagues indomethacin treatment, as mentioned above) is the risk of bony nonunion, as has been demonstrated when trochanteric osteotomy is necessary to remove the prosthesis during a revision procedure[4,49,54]. Rates of nonunion range from 12–30% after RT[4], with the highest in Lo et al., in which the authors noted that of the 6 patients in whom osteotomy was necessary, 2 developed a nonunion[54]. In non-irradiated trochanteric osteotomy, the rate of nonunion is diminished, occurring only 2–15% of the time[4]. Therefore some authors have advocated the use of shielding to prevent nonunion[9,50].

Finally, radiation dose to the testis is also a concern with the use of radiation prophylaxis. Animal studies have shown that reversible oligospermia can be induced with doses as low as 20 to 70 cGy, and doses of 120 cGy have been shown to cause permanent azoospermia[4]. The same study also measured effective testicular dose, showing an average of 25.1 cGy, with a 54% reduction to 11.3 cGy using a testicular shield. For this reason it is recommended that testicular shields always be used during this treatment and that patients be made aware of the risks involved with this treatment modality.

Regarding which of these treatments (ie NSAIDs or RT) is most effective, several trials have found both are effective means of prophylaxis following arthroplasty and trauma. They point out that clinical decisions should not be made based on efficacy but rather on factors such as availability, side effects and cost [55-57]. Strauss et al. (2008) recommend that, when accounting for efficacy and all costs associated with the use of NSAIDs versus RT (including patient disability due to HO) for prophylaxis, RT has the advantage due to a lower incidence of serious side effects[58]. This attitude is not supported universally, though several other trials have found an advantage in efficacy using RT rather than NSAID therapy[9,59]. Kölbl et al[59] conducted a trial in which 301 patients were randomized to either an NSAID treatment arm, a single 5 Gy fraction of RT or a 7 Gy fraction of RT. 113 patients were randomized to the NSAID arm, 95 to the 5 Gy arm and 93 to 7 Gy. The data from the study supported the 7 Gy therapy as being the most effective postoperative treatment schedule in prevention of clinically significant (Brooker 3 or 4) heterotopic ossification. A study by Pakos et al[9] had similar results, demonstrating a difference in effective HO prophylaxis for Brooker grade 3 and 4, albeit only a 1.2% absolute risk reduction.

### Combination Therapy

Another option for HO prophylaxis is to combine NSAIDs and RT. Both Pakos et al[9] and Piatek et al[60] found combination therapy to be effective, with Pakos' study of 54 patients having only 1 develop clinically significant HO, though with an overall incidence of 20.4%. This rate is noticeably higher than that produced by several other studies, notably Kölbl et al[59], in which the overall rate of HO formation was 11.6% in the 7 Gy RT arm, with no instances of Brooker grade 3 or 4 HO. Piatek's[60] study had only 1 of 24 patients to develop any HO. Very few studies have examined this treatment modality combination, and there is still a considerable amount of discussion regarding whether or not it is worth pursuing[61]. Though Piatek's study in particular is promising, larger trials must be implemented to discover the true utility or lack thereof of a combined prophylaxis protocol.

## Methods under investigation

Indomethacin use has led to the development of gastric ulcers and even gastrointestinal hemorrhage[24,26] in some patients, as well as bony nonunion[5,25,45] as a result of its systemic effects. Radiation therapy carries with it the risks of carcinogenesis[4,50], gonadal dysfunction[4], and bony nonunion[4,49,54], as with NSAID therapy. Since the development and use of NSAIDs and RT for HO prophylaxis, there have been several studies attempting to pinpoint new aspects of HO etiology and thus direct the development of new prophylactic modalities with increased efficacy and fewer side effects[1,2,62-64].

### Noggin

Though the exact etiology of HO is not yet fully understood, it is possible that the overexpression of certain bone morphogenetic proteins (BMPs) may have an influence on the formation of ectopic bone[2], most notably in skeletal muscle. BMP-4 in particular has been demonstrated to be elevated in diseases such as fibrodysplasia ossificans progressive (FOP), a disease characterized by the progressive ossification of soft tissue, especially muscle, tendons and ligaments[2,65]. A study by Kan et al[66] demonstrated experimentally that overexpression of BMP-4 via a particular gene promoter was able to induce a FOP-like phenotype.

In addition to the upregulation of BMPs, the downregulation of BMP antagonists such as Noggin, an extracellular peptide that binds and antagonizes BMPs, may play a role in the development of heterotopic bone[2,62,66-68]. Hannallah et al[2] have shown that retroviral delivery of Noggin into HO-predisposed muscle stem cells in mice (those expressing BMP-4) is able to reduce HO by 53–99% in a dose-dependent manner. This study also showed that tissue in which Noggin had been delivered exhibited an 83% decrease in area of HO following trauma (Achilles tenotomy). Aspenberg et al[62] also demonstrated the efficacy of Noggin delivery in prevention of HO, using both wild-type and engineered strains of the peptide and Glaser et al[68] demonstrated that direct delivery of Noggin as well as systemic delivery via adenovirus vector was effective in blocking BMP-4-induced heterotopic ossification. A unique study performed by Weber et al. utilized mutant forms of BMPs that worked in a similar way to BMP antagonists (like Noggin), but even more effectively[29].

Current research suggests that local and some forms of systemic delivery of Noggin is effective in combating formation of heterotopic bone in animal models. Our hope is that Noggin will prove capable of doing the same in humans, particularly at surgical sites, in light of the high rates of heterotopic bone formation following procedures such as hip and knee arthroplasty.

### Pulsed Electromagnetic Fields (PEMF)

Based on the assumption that local hypoxia has a role in the development of HO[69,70], a study by Kociæ et al[64] has suggested that the use of pulsed electromagnetic fields (PEMF) could prevent HO by increasing the rate of circulation and oxygenation of soft tissue surrounding a traumatic or surgical site. Group A consisted of 131 hips in 117 patients that were administered PEMF treatments starting 3 days postoperatively and interferential current starting approximately 14 days postoperatively. Group B also contained 131 hips in 117 patients, but these patients were treated with PEMF and interferential current, both starting 14 days postoperatively, as well as kinesitherapy. Group C consisted of 79 hips in 66 patients who had only kinesitherapy during postoperative rehabilitation. Group A had an overall HO incidence of 16.7%, Group B 43.5%, and Group C 50.6%. (Group A vs. B and vs. C both *p *<*0.05*) Brooker grade 3 and 4 was evident in none of the Group A patients, in 6.1% of Group B patients and in 26.6% of Group C patients.

### Free Radical Scavengers

Oxidative stress occurs when the production of Reactive Oxygen Species (also referred to as free radicals) are created at a faster rate than that at which they are eliminated[1,71]. Large quantities of free radicals are produced both as a result of the ischemia/reperfusion syndrome and the so-called disuse phenomenon. The ischemia/reperfusion syndrome is a result of isometric muscle contractions (secondary to muscle hypertonia and contractures) effectively occluding the muscle's arterial supply and inducing a state of ischemia. Patients may be started on an exercise regimen to aid in reperfusion of the tissue, simultaneously causing free radical generation[1].

Vanden Bossche describes well the disuse phenomenon. Muscle atrophy occurs largely as a result not of degradation of existing proteins, but of the decrease in protein synthesis, weakening repair mechanisms[1,71]. However, a reduction in antioxidants available to the tissue also plays a role, complicated by the release of myoglobin-derived iron in muscle, which is able to catalyze oxidative processes responsible for further protein damage[1]. Moreover, the metabolic rate of the muscle involved is also a consideration, with higher metabolism being associated with a higher rate of production of free radicals, and thus increased tissue damage[1,72]. Knowing that heterotopic bone formation is noted to be present following both of these conditions, Vanden Bossche et al[1] indirectly links the production and action of free radicals to the process of heterotopic ossification. Drawing on the theory of the involvement of hypoxia in the pathophysiology of HO, the study examined the use of allopurinol and N-acetylcysteine (A/A) as free radical scavengers to prevent ectopic bone formation. Using a rabbit model, they used 10 animals each in 4 different treatments: placebo, indomethacin, A/A free radical scavengers, or a combination of free radical scavengers and indomethacin. They immobilized the left hind leg of each animal to induce HO. This study demonstrated a significant difference (*p *<*0.05*) between the efficacy of indomethacin and the increased efficacy of A/A on prevention of HO, as well as a statistical difference between the A/A and placebo group. However, no statistical significance was noted between the indomethacin and placebo groups. Also of note was that there was not significant difference in efficacy between the A/A group and the combination A/A, indomethacin group.

## Conclusion

Though radiation therapy and NSAIDs remain the most widely used therapeutic modalities in the setting of post-surgical heterotopic ossification prophylaxis, it is evident that they bring with them many hazards and shortcomings. While they represent comparatively cheap and easily administered treatments, they are plagued with the propensity to cause bony nonunion, gastrointestinal disturbances, and in the case of radiation therapy, carcinogenesis. Though proponents of COX-2 selective NSAIDs have sought to correct many of these ills, there is as of yet not enough evidence of their safety for the authors to recommend their adoption without reservation. Noggin (a bone morphogenetic protein inhibitor), pulsed electromagnetic fields (PEMF), and free radical scavengers in the form of allopurinol and N-acetylcysteine are three new methods being evaluated to take the place of radiation and NSAID therapy as the predominant method of prophylaxis. While research into alternative prophylactic modalities is currently in its infancy, the three mentioned here have thus far shown promise for fewer associated side effects and better control of heterotopic bone formation. It is the hope of the authors that these modalities will be further developed and made available for routine use, leading to fewer post-surgical complications and better outcomes overall.

## Competing interests

The authors declare that they have no competing interests.

## Authors' contributions

EOB wrote the paper. QKK conceived and designed the paper and wrote the abstract. Both authors reviewed the literature, read and approved the final manuscript.

## Supplementary Material

Additional File 1 **Summary of selected studies of COX-2 selective NSAIDs from 2002 to 2008.** Click here for file
