# Supplementary material for: Prophylaxis of heterotopic ossification – an updated review
Source: J Orthop Surg Res. 2009 Apr 20;4:12. doi: 10.1186/1749-799X-4-12 (PMC2674414; doi:10.1186/1749-799X-4-12)
Supplement: Additional File 1 — Summary of selected studies of COX-2 selective NSAIDs from 2002 to 2008. [file 1749-799X-4-12-S1.doc]

Title: Summary of selected studies of COX-2 selective NSAIDs from 2002 to 2008

| Study | Participants/Intervention | Complications | Findings |
| --- | --- | --- | --- |
| Barthel et al., 2002[31]  Randomized controlled trial analyzing the use of indomethacin versus meloxicam for HO prophylaxis following hip arthroplasty | 272 patients  Indomethacin 100mg/day  Meloxicam 7.5mg/day  Meloxicam 15mg/day  All groups treated for 14 days | GI side effects: Indomethacin 4%  Meloxicam 2% | Indomethacin is better than meloxicam for postoperative HO prophylaxis, and that COX-1 may be involved, in addition to COX-2 in HO |
| Chen et al., 2006[41]  Meta-analysis of randomized controlled trials to compare risk of CV events when using COX-2 selective NSAIDs vs non-selective NSAIDs vs placebo | 40 randomized controlled trials, total of 88,116 patients |  | No significant difference in cerebrovascular events when using COX-2 selective NSAIDs compared with non-selective NSAIDs or placebo. Authors attribute increased thrombotic events to myocardial infarct |
| Grohs et al., 2007[32]  Randomized controlled trial on the use of rofecoxib vs indomethacin for HO prophylaxis following total hip arthroplasty | 96 patients  Indomethacin (50 pts) 100mg/day  Rofecoxib (46 pts) 25mg/day  Both groups treated 7 days | Indomethacin: 2 pts dropped out due to GI side effects | Rofecoxib (COX-2 selective NSAID) is similar in efficacy to indomethacin (non-selective NSAID) for HO prophylaxis following total hip arthroplasty |
| Legenstein et al, 2002[36]  Randomized controlled trial comparing meloxicam with indomethacin for postoperative HO prophylaxis | 116 patients  116 patients  Indomethacin (58 pts) 100mg/day  Meloxicam (58 pts) 7.5mg/day  Treatment for 12 days | Indomethacin: 1.4% nausea, 1.4% GI  symptoms, 5.3% headache, 2.8% vertigo  Meloxicam: 1.8% nausea, 1.8% GI symptoms, 20.1% headache, 10.5%  vertigo | Meloxicam (COX-2 selective NSAID) is similar to indomethacin for HO prophylaxis. Also, interesting that GI symptoms are higher with meloxicam. Also noted increased incidence of vertigo with meloxicam vs indomethacin. |
| Rahme et al, 2007[43]  Retrospective cohort study assessing benefits versus risks of COX-2 selective NSAIDs compared with non-selective NSAIDs and acetominophen | Person-years of exposure:  in non-aspirin users:  75,761 to acetaminophen, 42,671 to rofecoxib,  65,860 to celecoxib,  37,495 to non-selective NSAIDs  In aspirin users:  38,048 to acetaminophen, 14,671 to rofecoxib,  22,875 to celecoxib,  9 832 to non-selective NSAIDs |  | Among non-aspirin users, naproxen exhibits the highest risk for AMI/GI bleeding. For AMI/GI toxicity celecoxib was similar to acetaminophen and seemed to be better than rofecoxib and non-selective NSAIDs.  Among aspirin users, bothcelecoxib and naproxen seemed to be the least toxic. Thus, the COX-2 selective NSAID seems to exhibit the fewest side effects. |
| Saudan et al, 2007[34]  Randomized controlled trial comparing ibuprofen with meloxicam for postoperative HO prophylaxis | 250 patients  Ibuprofen (127 pts) 1200mg/day.  Meloxicam (123 pts) 400mg/day.  Treatment 10 days | Ibuprofen: 3 pts with GI  symptoms, 4 pts with renal impairment  Meloxicam: 1 pt with GI symptoms, 6 with renal impairments | Meloxicam demonstrated a 50% reduction in incidence of Grade I HO and 75% reduction in Grade II and III when compared to ibuprofen. No difference noted in incidence of GI or renal complications. |
| Van der Heide et al, 2004[35] Prospective cohort study comparing meloxicam and indomethacin for postoperative HO prophylaxis | 181 patients  Indomethacin (pts) 150mg/day  Meloxicam (pts) 15mg/day  Treatment for 7 days | Indomethacin:  2 pts with GI symptoms, 10 pts prolonged wound drainage  Meloxicam: 1 pt with GI symptoms, 6 pts with prolonged wound drainage | The effects of meloxicam are comparable to those of indomethacin for the prevention of HO postoperatively, albeit with fewer side effects. |
| Van der Heide et al, 2007[37]  Randomized controlled trial comparing rofecoxib and indomethacin for postoperative HO prophylaxis | 186 patients:  Indomethacin (89 pts) 150 mg/day  Rofecoxib (85 pts) 50 mg/day  Treatment for 7 days | Both treatments had 6 patients (12 total) to prematurely withdraw from the study due to nausea and vomiting. | The effects of rofecoxib are similar to those of indomethacin for the prevention of HO postoperatively. This suggests the involvement of COX-2 in the etiology of HO. |
| Van der Linden et al, 2008[42]  Severe cardiovascular and gastrointestinal events among users of selective and non-selective NSAIDs | Hospitalization data of > 2 million patients in Netherlands, subjects with first hospitalization for AMI, CV and GI events were identified. Use of COX-2-inhibitors and non-selective NSAIDs was classified into remote, recent and current. |  | AMI and CV risk was similarly increased with individual use of COX-2 selective NSAIDs and non-selective NSAIDs, whereas GI risk was demonstrated to be higher with naproxen and diclofenac. |

AMI - acute myocardial infarction; GI – gastrointestinal; CV - cardiovascular
